# Supplementary material for: 11p15 Epimutations in Pediatric Embryonic Tumors: Insights from a Methylome Analysis
Source: Cancers (Basel). 2023 Aug 25;15(17):4256. doi: 10.3390/cancers15174256 (PMC10486592; doi:10.3390/cancers15174256)
Supplement: Supplementary file 1 [file cancers-15-04256-s001.zip › cancers-2501311-supplementary.pdf]

Supplemental Table S1. DNA methylation levels of ICR1 and ICR2 for each tumor analyzed by EPIC array, MS-MLPA and cfDNA experiments.

Sample	MS-MLPA ICR1 (% of methylation)	EPIC ICR1 (% of methylation)	cfDNA ICR1 (% of methylation)	MS-MLPA ICR2 (% of methylation)	EPIC ICR2 (% of methylation)	Cluster at Heatmap
ES_28	45	59	51	51	52	2
WT_02	52	57	52	55	50	2
HB_40	55	60	56	55	42	2
NB_24	48	62	80	55	64	2
WT_10	57	55	49	58	56	2
CCSK_38	47	55	67	59	56	2
NB_23	51	56	41	80	62	2
ES_31	50	57	72	51	51	4
CCSK_37	54	57	58	53	54	4
ES_29	53	57	69	54	48	4
NB_19	52	56	59	55	51	4
ES_30	54	56	40	58	47	4
NB_17	55	57	57	59	51	4
NB_25	47	53	55	64	52	4
WT_01	85	92	84	5	2	3.1
WT_13	98	89	70	6	2	3.1
WT_11	90	81	71	7	12	3.1
NB_22	124	90	80	9	1	3.1
WT_07	99	92	78	10	2	3.1
HB_39	95	84	79	11	26	3.1
WT_03	92	85	83	17	12	3.1
ERMS_33	78	83	59	22	42	3.1
WT_05	107	84	78	58	48	3.2
WT_09	102	76	94	62	60	3.2
NB_21	114	79	81	62	54	3.2
NB_20	64	61	79	33	40	3.3
WT_14	79	68	51	35	43	3.3

NB_26	60	57	74	41	41	3.3
WT_04	75	66	89	59	51	3.3

Dark pink indicates cases in which peripheral blood samples were collected during follow-up. For MS-MLPA and EPIC experiments, the average of methylation values are shown.

**ICR1**	**CRITERIA**	
MS-MLPA	41-59	NORMAL
	>60	GOM
	<40	LOM
EPIC		
	40 - 60	NORMAL
	>60	GOM
	<40	LOM
cfDNA	41-62	NORMAL
	>62	GOM
	<41	LOM
**ICR2**	**CRITERIA**	
MS-MLPA	>44	NORMAL
	<44	LOM
EPIC		
	40 - 60	NORMAL
	>60	GOM
	<40	LOM
